# Investigation of Silicone-Containing Semisolid in Situ Film-Forming Systems Using QbD Tools

**DOI:** 10.3390/pharmaceutics11120660

**Published:** 2019-12-07

**Authors:** Nikolett Kis, Anita Kovács, Mária Budai-Szűcs, Attila Gácsi, Erzsébet Csányi, Ildikó Csóka, Szilvia Berkó

**Affiliations:** Institute of Pharmaceutical Technology and Regulatory Affairs, Faculty of Pharmacy, University of Szeged, 6720 Szeged, Hungary; nikolet951@gmail.com (N.K.); anita.kovacs@pharm.u-szeged.hu (A.K.); maria.szucs@pharm.u-szeged.hu (M.B.-S.); gacsi.attila@pharm.u-szeged.hu (A.G.); csanyi@pharm.u-szeged.hu (E.C.); csoka@pharm.u-szeged.hu (I.C.)

**Keywords:** in situ dermal film, film forming system, quality by design, silicones

## Abstract

The aim of our research work was to develop dermally applicable, semisolid film-forming systems (FFSs) containing silicones, which form a film on the skin in situ, with suitable mechanical properties for skin application. FFSs were developed and investigated by means of the Quality by Design (QbD) methodology. With this QbD approach, the initial risk assessment defines the critical quality attributes (CQAs), the critical material attributes (CMAs) and the critical process parameters (CPPs) to ensure the required quality. Different semisolid systems were formed with or without silicones. During the initial risk assessment, three CQAs, namely skin adhesion, film flexibility and burst strength, were found to be critical attributes, while film appearance, film integrity and the drying time of the semisolid system, were found to be medium attributes. These parameters were investigated. The initial risk assessment also showed that there are three high CMAs: the type of silicones, film-forming excipients, drying excipients, and that there was one medium CMA: viscosity-enhancing excipients. Based on our results, the silicone content had a great effect on the film-forming systems. Different silicones affected the mechanical properties of the films in varying ways, decreased the drying time and showed promising results regarding the drying mechanism.

## 1. Introduction

The dermal route of drugs has high potential in the health care system. One of the most important advantages of this route is the possibility to achieve a high concentration of the drug on the application site, which is promising for local anesthesia, arthritis or acne treatment. It gives good patient compliance, as it is a comfortable and painless administration route.

On the other hand, it is a great challenge to provide penetration through the skin because this organ is the first protection line against external impacts [1]. The skin has three functional layers: the epidermis, the dermis and the subcutis [2]. The outermost layer of the epidermis is the stratum corneum (SC), which is the most important layer to protect the skin. It has many functions, such as, to inhibit excessive water loss to ensure optimal skin hydration, and to defend the body from microbial pathogens, such as bacteria, and from various toxins as well [3].

The amount of active ingredients penetrating into the SC depends upon the properties of the drug, the physiological parameters of the skin, and the applied drug delivery system. From the aspects of pharmaceutical technology, one of the main tasks is to ensure and improve the penetration of the active ingredients into the desired skin layer. There are some innovative dermal drug delivery systems, such as the in situ film-forming system, which can be a promising choice to achieve higher drug penetration through the skin with good patient adherence.

In situ film-forming systems (FFSs) are new alternative, semisolid drug delivery systems [4,5] that produce a film in situ, after application onto the skin surface. They are composed of volatile and non-volatile components. After the application, the volatile component(s) evaporate(s), leaving an in situ film upon the skin. The non-volatile components make a matrix, encompass the drug and provide the transport through the stratum corneum [6]. With the evaporation of the volatile components, the drug concentration increases on the skin surface, creating a supersaturated system. Supersaturation increases drug penetration through the skin, without modifying the skin barrier [6]. Compared with transdermal patches and conventional semisolid dosage forms, FFSs possess all their advantages. FFS preparations can be retained for a longer time than conventional semisolid preparations, thanks to their good adhesion to the skin. They are almost invisible, dry fast, and after drying they have a non-sticky, non-greasy feeling, and due to their flexibility they are easily removable [7]. FFSs could be a promising choice for dermal application, for example in acne therapy or local anesthesia to improve the patient’s adherence.

When designing a film-forming system, it is important to choose appropriate components to ensure the formation of an optimal film product. The critical point of the compositions is the appropriate volatile and non-volatile components and their features. Film-forming agents, drying excipients and viscosity-enhancing agents are essential components to get a film product [8].

Silicones are popular ingredients in the pharmaceutical and cosmetics industries. In the pharmaceutical application, the most commonly used silicones are cyclomethicone and dimethicone [9,10]. In the case of in situ film formation, silicones can influence the properties of the film, and thus skin penetration, too. Cyclomethicone is a volatile silicone which can influence the drying properties of FFSs. Dimethicone is a non-volatile silicone that can improve the quality and the performance of dermal preparations, as it gives a “silky touch” feeling, has a good protective effect and also softens the skin. They are safe to use, provide an aesthetic appearance for the product and are resistant against water washing [11]. The silicone molecules can make a semi-occlusive formulation, which can influence the penetration of active ingredients into the SC.

In the development of new pharmaceutical formulations, appropriate quality can be ensured with the Quality by Design (QbD) concept [12]. It is a holistic, systematic approach that begins with predefined targets, and emphasizes product and process understanding, as well as process control, based upon quality risk management [12,13,14]. The QbD concept involves identifying the quality target product profile (QTPP), the critical material attributes (CMAs), the critical process parameters (CPPs) and the critical quality attributes (CQAs) of a product at the beginning of its development [15]. These factors are screened with risk assessment within those quality tools. Several quality tools can be found in the International Council for Harmonization (ICH) guideline Q9 (e.g., Ishikawa diagram, Pareto analysis, risk estimate matrix (REM), etc.). The critical parameters of the formulation are defined based upon risk assessment [16,17]. During in situ FFS development, the QbD method is a novel approach which improves the product quality by understanding formulation parameters.

The aim of our research work was to develop a dermally applicable, silicone-containing semisolid in situ FFS on the basis of the QbD approach. Our study focuses on the determination of critical quality parameters, which have the highest influence on the formulation, using QbD tools. The most critical parameters are required to be examined, and the effect of different silicones on these parameters must be compared.

## 2. Materials and Methods

### 2.1. Materials

Poly(vinyl alcohol) (PVA) (87%–90% hydrolyzed, M_w_ = 30,000–70,000 g/mol) was from Sigma-Aldrich (Budapest, Hungary). Ethanol (96 per centum, Ph. Eur. 9.) was obtained from Molar Chemicals Ltd. (Budapest, Hungary). Xantural^®^ 180 Xanthan Gum was provided by CP Kelco A Huber Company (Atlanta, GA, USA). ST-Cyclomethicone 5-NF, Dimethiconol Blend 20, ST Elastomer 10 and 7-3101 Elastomer Blend HIP Emulsion were kindly provided by Dow Corning (Midland, MI, USA). Purified and deionized water was used (Milli-Q system, Millipore, Milford, MA, USA). 

Methyl parahydroxybenzoate (Ph. Eur. 9.) was supplied by Molar Chemicals Ltd. (Budapest, Hungary). Salvequick^®^ sticking plaster was obtained from Orkla Care AB (Solna, Sweden). PVA-based artificial skin was from Tattoo machine Webshop (© 2018, Mátészalka, Hungary).

### 2.2. Quality by Design Methodology

#### 2.2.1. Definition of TPP and QTPP

The first step of development by the Quality by Design (QbD) approach is to define the Target Product Profile (TPP), such as the route of administration, dosage form and strength, etc. [18]. In order to reach the quality properties, we need to summarize the quality target product profile (QTPP). The QTPP includes quality, efficiency and safety, with the consideration of appearance, physical properties, homogeneity, etc. depending on the dosage form [18].

#### 2.2.2. Definition of CQA, CMA, CPP

When QTPPs have been defined, the second step of the QbD-based development is to determine the critical quality attributes (CQAs) which have to be ensured to achieve the required final quality of the product. The CQAs are derived from the QTPP. The CQAs are physical, chemical or microbiological properties within an appropriate range to ensure the desired product quality. The CQAs may include pH, homogeneity, viscosity, adhesion properties, etc., depending on the dosage form, such as semisolid systems. The next step of the QbD-based development is to define the critical material attributes (CMAs) and critical process parameters (CPPs) that may influence product CQAs. These factors include properties of the excipients (e.g., polymer and surfactant), properties of the drug and process parameters (e.g., homogenization time and temperature) [19,20,21].

#### 2.2.3. Risk Assessment: Quality Tools

**Figure 1:**
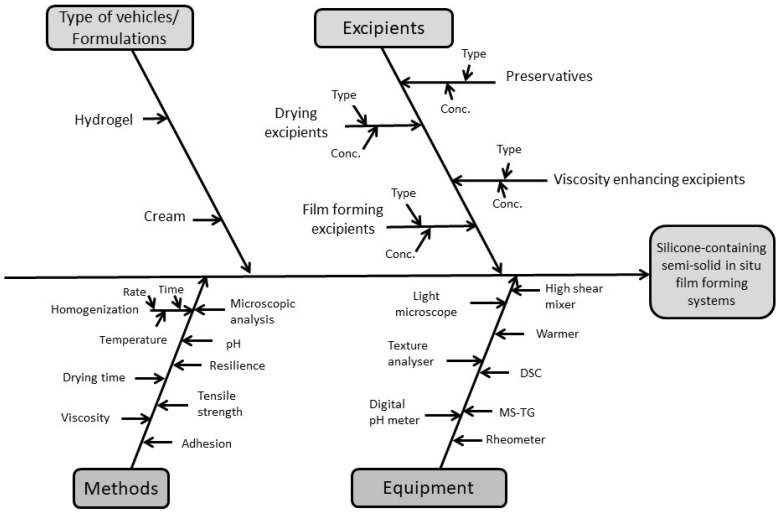
Ishikawa diagram of material attributes and process parameters of film-forming systems (FFSs).

Risk assessment is the base of the QbD concept. Risk assessment involves the use of quality tools, which help improve the quality of products and processes. In our research, risk assessment started with one popular basic quality tools method called the Ishikawa diagram. This method is one of the “cause and effect” diagram types, which helps to collect the effect and the possible causes influencing quality. The essence of the method is that the causes are ordered in an organized system. Not only the major causes, but also the more detailed factors, are identified [22]. The Ishikawa diagram summarizes materials and process parameters to help the development of a semisolid film-forming system (Figure 1).

Critical parameters were determined by Pareto analysis, also called ABC analysis [21]. A-items are the high-risk parameters, B-items are the medium-risk parameters and C-items are the low-risk parameters [17]. A risk estimate matrix (REM) was used during risk assessment to define the level of the risk parameters. The LeanQbD™ software (QbD Works LLC, Fremont, CA, USA) was used for the risk assessment.

### 2.3. Preparation of FFSs

Three different kinds of FFSs were prepared. The composition of the different formulations can be found in Table 1. One of the formulations did not contain silicones (F1), but the other two FFSs contained different volatile and non-volatile silicone components (F2, F3). The function of volatile silicone (ST-Cyclomethicone 5-NF) was to ensure the fast drying of the film. The other type of silicones was that type of non-volatile silicones, such as Dimethiconol Blend 20, ST-Elastomer 10 and 7-3101 Elastomer Blend HIP Emulsion. They have different functions in the formulations, they are film-forming excipients, and furthermore can influence the appearance of the film, give a “silky touch” feeling, have a good protective effect on the skin, and soften it. F2 included 25% of silicones, while F3 contained 50% of silicones in the composition. The amount of volatile silicone was the same in both formulations. PVA and Xanthan gum are film-forming and viscosity-increasing excipients in the formulations. Finally, the ethanol content helps the film to dry and ensures the required drying time together with volatile silicone [7,23].

During the preparation, PVA was dissolved in purified water at 80 °C under continuous mixing. Ethanol 96% was added at room temperature. After that, Xanthan gum was added to provide optimal consistency. Finally, the silicones were added one by one slowly and mixed with a high shear mixer.

### 2.4. Investigation of the Mechanical Parameters of the Films

The mechanical properties of the films were investigated with a TA.XT plus Texture Analyzer (Stable Micro Systems Ltd., Vienna Court, Lammas Road, Godalming, Surrey, UK. GU7 1YL) [24]. The instrument has different accessories, and depending on the formulation, different attributes of the film can be characterized. Film Support Rig and 90 Degree Peel Rig equipment were used during the experiment.

#### 2.4.1. Measurement of Skin Adhesion

Skin adhesion studies investigate the force needed to separate the film from the skin surface. For these measurements, a film with a surface of 10 × 2 cm was used. 90 Degree Peel Rig equipment was used to measure the initial and the mean peeling force which was needed to separate the film from the artificial skin [25]. The test speed was 5 mm/s and the distance was 50 mm. During the evaluation, the mean peel force was averaged from 10 mm to 45 mm.

#### 2.4.2. Measurement of Film Flexibility

The flexibility of the film can have an effect on skin feeling (and thus on patient adherence), and it can help the formulation to remain on the skin surface. It was analyzed by the resilience value and the mean force at the target distance. The free film was also placed into the film supporting rig [26]. A compression test mode was used where the test speed, force and target distance were 0.5 mm/s, 100 g and 1 mm, respectively. Force at target distance (N) and resilience (%) were detected. The detected curve had a maximum peak when the test reached the target distance. Resilience is the ratio of the areas after and before the maximum peak. The measured film resilience values were compared with the resilience of the heat-separated human epidermis. The preparation of this heat-separated epidermis was based upon a procedure reported by Kligman and Christophers [27]. 

#### 2.4.3. Measurement of Film Burst Strength

Burst strength is the force needed to rupture the film from the skin surface, while distance at burst is the maximum distance of the deformation before the rupture. Circles with a diameter of 22 cm were cut from the peeled free film and placed into the film support rig [26]. A compression test mode was used where the test speed, force and distance were 1 mm/sec, 100 g and 10 mm, respectively. Burst strength (N) and distance at burst (mm) of the films were measured.

### 2.5. Investigation of Film Integrity and Appeariance 

For the measurement, 0.2 g of each formulation was deposited on a 3 × 3 cm surface of the PVA-based artificial skin, left there until it was completely dry, and then the properties were investigated visually.

### 2.6. Investigation of the Drying Time of FFSs

Drying time is an important parameter of the FFSs. If the preparations need less time to dry, patient compliance is better. After drying, a compact flexible film layer is formed, which is not greasy and does not smear on the skin. During the experiment, the preparation was placed onto the PVA-based artificial skin. After the top of the layer seemed dry, a microscope slide was placed on the top of the formulation without pressure. If the formulation did not leave a mark on the slide, it meant that the film was dry. This was the drying time of the film. If the film was not dry, the test was repeated until we got a dry film [28].

The drying mechanism of the different FFSs was measured by DSC (Mettler-Toledo DSC 821e, Columbus, OH, USA). The measurement was performed in an argon and nitrogen atmosphere. 15–20 mg of samples was laid in 40-μL aluminum pans and small holes were made on the tops. The temperature was increased from 25 °C to 150 °C by 10 °C per minute.

### 2.7. Statistical Analysis

The one-way analysis of variance (ANOVA—Dunnett) with Prism 8 for Windows software (GraphPad Software Inc., La Jolla, CA, USA) was used to analyze the results statistically. The differences were significant if *p* < 0.0001**** versus the control [18].

## 3. Results & Discussion

### 3.1. Determination of QTPP and CQAs for In Situ FFSs

The QTPP of the FFS-containing silicones includes the route of administration, dosage form, site of activity, appearance of drug delivery system, stability, silicone content, packaging material type and mechanical properties of the film for skin application. The properties of the semisolid system and the formed film depend on the type and attributes of the applied excipients. The silicone content can influence the properties of the FFS, such as the mechanical and drying properties, and the appearance of the preparation. The CQAs are defined from the QTPPs. The CQAs were determined with the consideration of the characteristics of the semisolid system and the formed film, too. On the one hand, the properties of the semisolid system include physical properties, viscosity, homogeneity, pH, skin feeling, drying time, physical, chemical and microbiological stability. On the other hand, the formed film has properties like film appearance, burst strength, skin adhesion, film flexibility and integrity. Table 2 and Table 3 show the QTPP and CQA parameters with their targets and their justifications.

### 3.2. Initial Risk Assessment

Risk assessment refers to the estimate of the risks related to the semisolid in situ FFS containing silicone. Risk assessment tools can be used to identify and rank parameters (e.g., process, input materials) with the potential to have an impact upon product quality. After the determination of QTPPs and CQAs, the following step is to determine critical material attributes (CMAs) and critical process parameters (CPPs) of the FFS, which are shown in Table 4.

**Figure 2:**
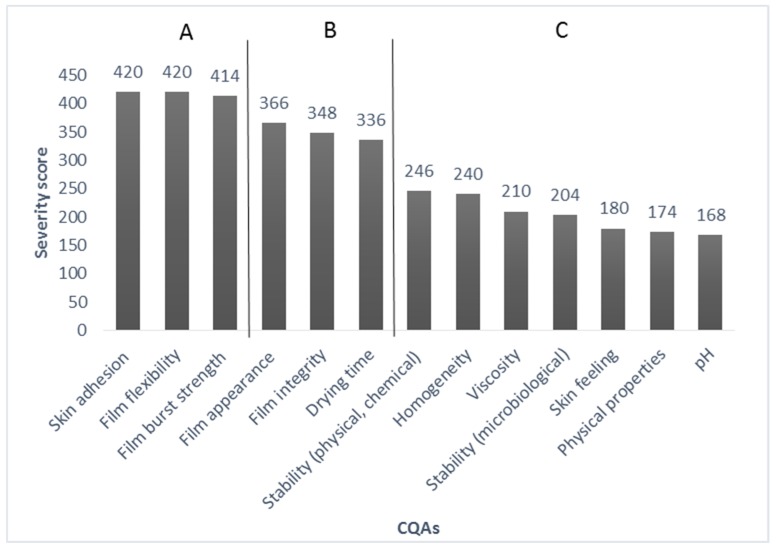
Pareto chart of CQA parameters.

The risk estimate matrix (REM) includes the connection between the parameters to recognize the critical quality parameters of the target product [30]. The first REM shows the relationship between QTPPs and CQAs (Table 5). A three-step scale was used to evaluate the probability relationship between the parameters: Low (low risk parameters), Medium (medium risk parameters), High (high risk parameters) were the optional levels. For the probability rating a 1(low)-3(medium)-9 (high) scale was used. These are the parameters that influence product quality: physical properties (5%), viscosity and homogeneity (both 6%), pH and skin feeling (both 5%), drying time (9%), physical and chemical stability (7%), microbiological stability (5%), film appearance (10%), film burst strength, skin adhesion and film flexibility (both 11%) and film integrity (9%). Based on the results of REM, a Pareto chart (Figure 2) was created showing the severity scores of the CQAs. The chart draws attention to the parameters which have a critical effect on quality during the developing process [31].

The results show that three film properties: skin adhesion, film flexibility and burst strength are the most critical parameters with the highest severity score (>400), called Category A, during the development. The next category of severity scores (300–400) is Category B, which includes film appearance, film integrity and the drying time of the semisolid system. The third category of severity scores is Category C (below 300), which has a low impact during development. High (Category A) and medium (Category B) risk parameters, which potentially have an effect on the quality of semisolid in situ FFSs, are investigated in this research work.

**Figure 3:**
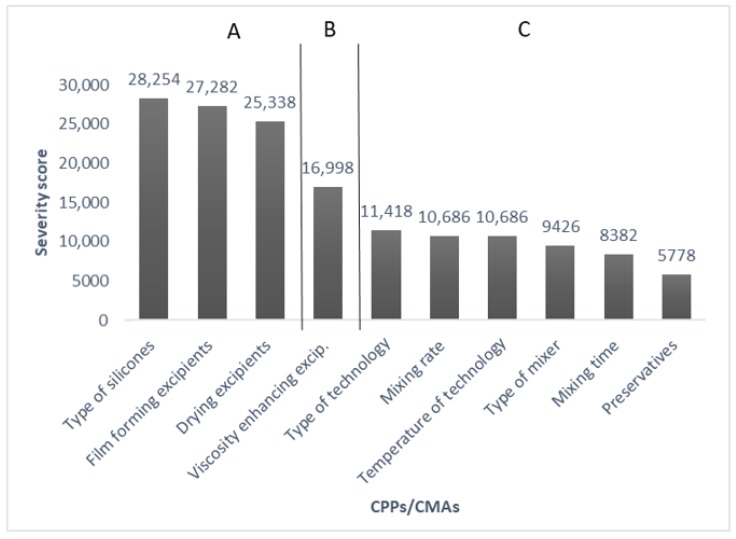
Pareto chart of CPPs/CMAs.

The second REM (Table 6) shows the relationship between CPPs, CQAs and CMAs. The same scale was used for evaluation (low, medium, high). These are the critical process parameters: mixing rate and time, type of mixer, temperature and type of technology. The critical material parameters are viscosity enhancing excipients, preservatives, drying excipients, film-forming excipients and type of silicones. Based on the results of the initial risk assessment (Figure 3), there are three groups of parameters considering the risk level. Critical parameters in Category A are some material parameters: type of silicones, film-forming excipients, drying excipients with the highest severity score (>20,000). They have the greatest impact during formulation. Category B includes viscosity-enhancing excipients with a medium severity score (15,000–20,000), which also have a considerable effect on quality. These are the investigated parameters during the experiment. Category C has the lowest impact during development (>15,000).

To sum it up, the FFSs have been planned to ensure the required mechanical properties for dermal application using QbD tools. This approach allows us to establish the minimum number of experiments to reduce costs and save time.

The initial risk assessment defined the CMA and CPP parameters to ensure the required CQAs. Based on the results, the initial risk assessment showed that there were three highly critical material parameters for CQAs that were the type of silicones, film-forming excipients and drying excipients, and one medium critical material parameter for CQAs, namely viscosity-enhancing excipients. The process parameters were not found to be highly critical parameters in this development.

Three CQAs, namely skin adhesion, film flexibility and burst strength, were found to be critical attributes for the FFS. Furthermore, three CQAs, namely film appearance, film integrity and the drying time of the semisolid system, were found to be attributes of medium influence. These parameters were investigated during this research work.

### 3.3. Skin Adhesion

Skin adhesion studies investigate the force needed to separate the film from the skin surface. This parameter is very important because good adhesion ensures a longer residence time, but on the other hand, patients prefer to remove the film without pain, therefore adhesion should be within an optimum range.

**Figure 4:**
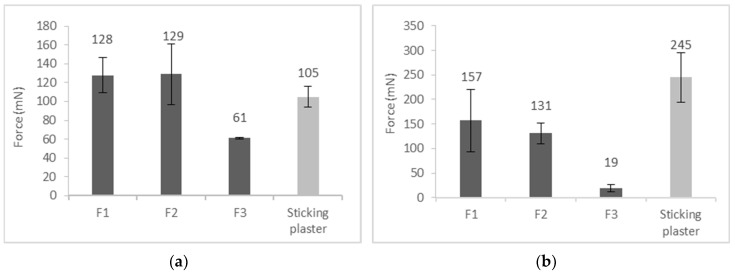
Skin adhesion: (**a**) Initial peel; (**b**) Mean peel.

During the experiment, three films were compared with a conventional sticking plaster. The initial peel and the mean peel forces were measured (Figure 4). The initial peel represents the force to start pulling off the film, while the mean peeling force is the average of the measured values during the experiment. Film F1 without silicone (128 mN) and Film F2 with silicones (129 mN) needed nearly as much force to start pulling off the film as the sticking plaster (105 mN), so they met the requirements of risk assessment (100–200 mN). In contrast, Film F3 with silicones (61 mN) was easier to start pulling off.

In the case of the initial peel, the elongation of a film fixed in the upper sample holder of the instrument resulted in higher adhesion force values compared with the more rigid sticking plaster. This difference and tendency changed after the initial peel; in the second part of peeling, clear adhesion force dominated.

Films F1 (157 mN) and F2 (131 mN) also meet the recommended mean peel requirements of the CQA (100–500 mN). The low value of Film F3 (19 mN) suggests an easy peeling off from the skin, and thus a short residence time. During the further development of Film F3, an effort should be made to increase the adhesiveness of the formulation. In summary, the type and the amount of silicones have remarkable influence on the adhesion of the films to the skin.

### 3.4. Film Flexibility (Resilience)

One of the most important mechanical properties of films is flexibility, because it can have an effect on skin feeling, and it can help the formulation to remain on the skin surface. If the film is too rigid, the movement of the skin surface can induce the development of peeling force, and thus film separation from the surface. Therefore, during development skin-like flexibility is recommended as a target value.

During our experiments, the flexibility of three films was compared with each other and with that of the heat-separated human epidermis. In the CQA an optimal flexibility range (above 25%) was established. The flexibility of the system was characterized by two parameters: the first is the mean force at target distance, which shows the resistance of the films during stretching, and the second is the resilience value. The mean force value at target distance can predict the deformability of the system; when this force is low, the system is very deformable (minimal force can induce deformation in the system). The resilience value shows the recovery of the sample from deformation, this parameter can provide information on product elasticity [32].

**Figure 5:**
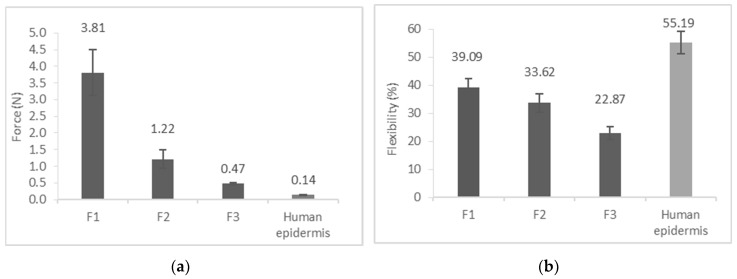
Film flexibility: (**a**) Mean force at target distance; (**b**) Resilience.

As the results show (Figure 5), Film F1 and Film F2 are in the optimal range of resilience values, which means that these systems can follow and accommodate to the skin movement during the application. Film F1 (39.1%) has resilience similar to that of the human epidermis (55.2%), and it can be inferred from the mean force at target distance that this system has more elastic properties. Film F3 (22.9%) has the lowest resilience, which means it has the weakest elasticity.

### 3.5. Film Burst Strength

**Figure 6:**
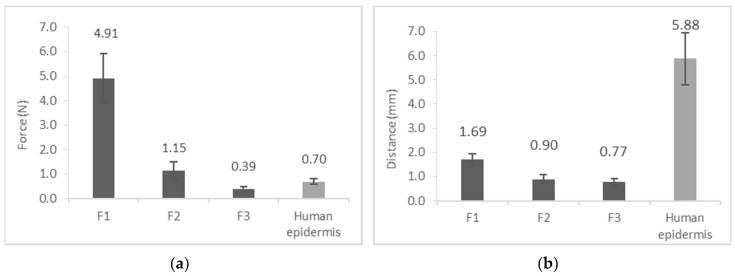
Film burst strength: (**a**) Burst strength; (**b**) Distance at burst.

During these measurements, the stiffness of the three films were evaluated and compared with each other. Burst strength is the force needed to rupture the film, while distance at burst is the maximum distance of the deformation before the rupture. Film F1 (4.91 N) has the highest film burst strength, which means that this film is the hardest to break. It is also slightly distensible, Films F2 (1.15 N) and F3 (0.39 N) need lower force to burst, which means that silicones decrease the stiffness of the films (Figure 6).

### 3.6. Film Appearance

**Figure 7:**
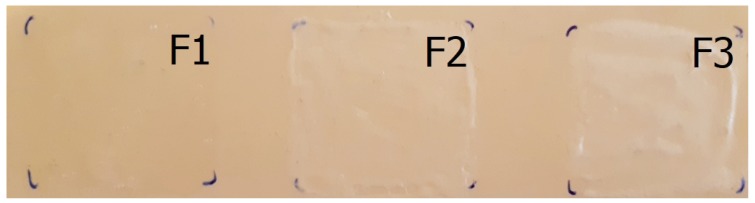
Film appearance.

The preparations formed translucent, homogeneous, compact films as Figure 7 shows. The film was whiter after drying in the case of Film F3. The films containing silicones were softer and not as rigid as Film F1. All three films met the requirements, where Films F1 and F2 are optimal if the target is long-term translucent usability.

### 3.7. Film Integrity

**Figure 8:**
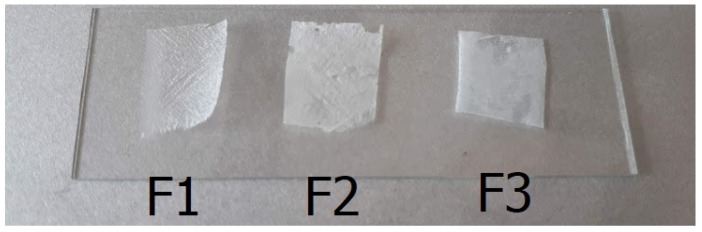
Film integrity.

All three formulations formed compact films on the skin surface, and the films could be removed in one piece from the skin (Figure 8). The films met the requirements, but Film F1 was more compact than films containing silicones.

### 3.8. Drying Time of FFSs

**Figure 9:**
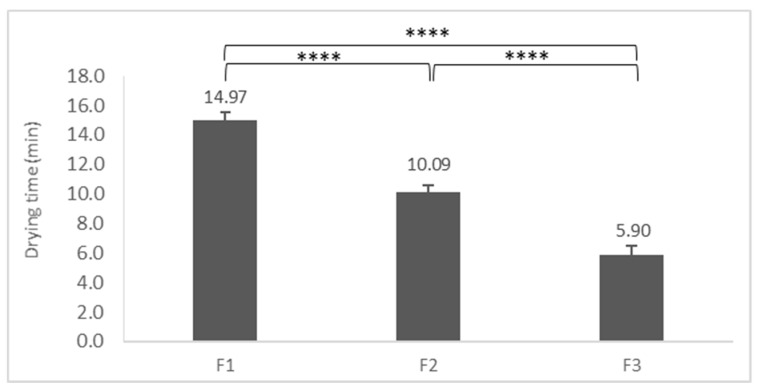
Drying time; (*p* < 0.0001****).

The results show (Figure 9) that Film F1 had the longest drying time. The film needed almost 15 min to get dry on the PVA-based artificial skin. In the case of systems containing silicones, drying time decreased. The drying time of Film F2 was about 10 min. With almost 6 min, the drying attribution of Film F3 was significantly the fastest.

As for Film F1, the whole thickness of the film was dry during the drying time. In the case of F3, the structure of the film was different; the top of the film was dry in 6 min, but the inner layers stayed soft and wet, which can predict better and longer penetration through the skin.

**Figure 10:**
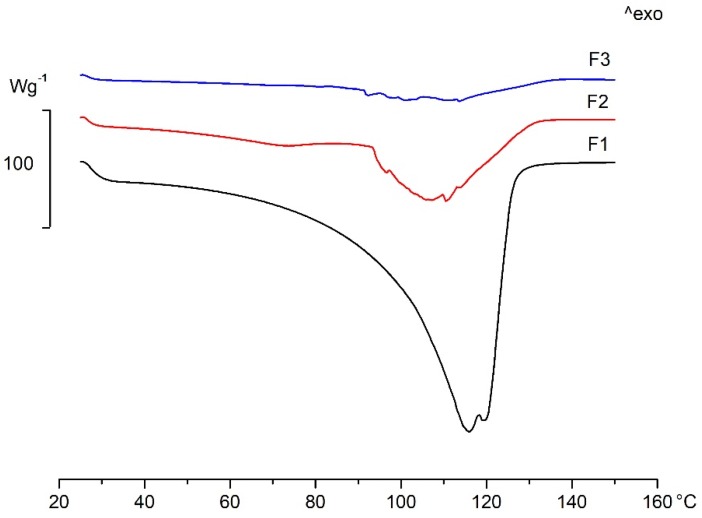
DSC curves of different FFSs.

In order to prove the different drying time and drying mechanism of the FFSs, DSC measurements were performed (Mettler-Toledo DSC 821e, Columbus, OH, USA). The evaporation mechanism of solvent and volatile components from the FFSs has an effect on the drying of these systems and thus on the final characteristics of the films. Figure 10 shows the DSC curves of the different FFSs, and the DSC data can be found in Table 7. F1 contains water as its solvent, and ethanol as a volatile component. During the examination, one broad endothermic peak appeared on the curve (107 °C), which can be identified as the evaporation of water and ethanol simultaneously. In contrast, there are two peaks on the curve of F2, where the first peak (at 71 °C) corresponds to the volatile silicone (cyclomethicone has a flash point at around 77 °C [33]), while the second peak (at 107 °C) indicates the evaporation of water and ethanol together like in the case of F1. Similarly to F2, F3 also shows two peaks during drying, but the evaporation of the volatile components is not as separated as in the previous case (F2). 

The peak of the evaporated cyclomethicone (at 91.5 °C), and that of water and ethanol (at 112.6 °C) shifted to higher temperatures and overlapped with each other, which can mean a prolonged drying time.

When we consider the shape of the curves, it can be clearly seen that the run-off of the endothermic peak is not so smooth for F2 and F3, which can indicate evaporation hampered by a film layer on the top of the sample. This hypothesis is in accordance with the visual observation of film drying where a film layer formed on the top of the sample, while the deeper layers remained wet.

Summarizing the results, the silicone content accelerates drying time and influences the drying mechanism.

## 4. Conclusions

In the present work, dermally applicable, semisolid FFSs containing silicones were developed with the QbD approach, which forms a film on the skin with the expected mechanical properties.

Based on the results of initial risk assessment, three compositions were formed and investigated. Initial risk assessment defined the CMAs and the CPPs to ensure the required CQAs. During the initial risk assessment, three CQAs, namely skin adhesion, film flexibility and burst strength, were found to be highly critical attributes, and three CQAs, namely film appearance, film integrity and the drying time of the semisolid system, were found to be medium critical attributes in the development of FFSs. These parameters were investigated. The initial risk assessment also showed that there are three material parameters, namely type of silicones, film-forming excipients and drying excipients, which were highly critical parameters for the CQAs, while viscosity enhancing excipients had a medium impact on the CQAs.

The texture of FFS films is an important factor during development [34]. The physical properties of the film highly influence usability, patient compliance and efficacy. In the case of FFSs, the right burst strength ensures the resistance of the film on the skin surface. Good skin adhesion increases the residence time and thus the better bioavailability of the drug, but the easy removability of the film is also important. The resilience value of the system can be a critical factor as well, because if it is similar to skin flexibility, the system can follow the skin’s movement and provides better skin sensation.

During the experiments, important findings were made (Table 8). The film properties showed that all three compositions were appropriate regarding appearance and integrity. The films were translucent and homogenous, and could be removed in one piece. The texture of the films showed different results. Film F1 had the highest skin adhesion and flexibility (resilient), and the burst strength showed that this film needed the highest force to burst. Due to the silicone content, softer but less resistant films were obtained. Film F2 showed closer values to Film F1. As for drying time, Film F3 gave the most promising results because of its different drying mechanism, which can predict higher drug penetration through the skin in the further development of FFSs-containing drugs.

In summary, silicone content has a great effect upon the properties of the films. Different silicones affect the mechanical properties of films in different ways. Non-volatile silicones soften the films, thereby decreasing the mechanical attributes, while volatile silicone helps to form films and accelerate drying. Based on these results, the proper FFS-containing silicone could be chosen, which can function as an applicable drug delivery system in the case of any development of FFS containing different APIs.

## Figures and Tables

**Table 1:** Composition of different formulations.

Composition	Function of the Excipient	F1	F2	F3
Purified water with methyl parahydroxybenzoate	Solvent with preservatives	+	+	+
Ethanol 96%	Drying	+	+	+
PVA	Film forming, Viscosity enhancing	+	+	+
Xanthan gum	Film forming, Viscosity enhancing	+	+	-
Dimethiconol Blend 20	Film forming	-	+	-
ST-Elastomer 10	Film forming	-	+	-
7-3101 Elastomer Blend HIP Emulsion	Film forming	-	-	+
ST-Cyclometicone 5NF	Drying excipient	-	+	+

**Table 2:** Quality target product profiles (QTPPs) of the film-forming system (FFS).

QTPP	Target	Justification
Route of administration	Dermal	Dermal delivery is an opportunity to achieve a high concentration of the drugs on the application site and to avoid systemic side effects. Furthermore, it is a non-invasive and painless administration route, resulting in high patient compliance.
Dosage form	Semisolid in situ film-forming system	Suitable for dermal and transdermal application, it has a longer residence time and prevents smearing. Dermatological treatment may require less frequent dosing of the FFS [7].
Site of activity	Deeper layer of the skin	The dermal drug delivery system ensures penetration through the skin as deep as the therapeutic effects require.
Appearance of semisolid system	Transparent or white, homogeneous	To ensure the aesthetic appearance of the film, the semisolid system has to show similar properties. To increase patient compliance [7].
Stability (physical, chemical)	Homogeneous; in the formulation there is no visible sign of instability	To ensure applicability, stability is a critical point of formulation. Appearance change, phase separation, pH change and viscosity change are stability issues, which can inhibit usability.
Silicone content	Film formation, fast-drying, silky touch	To change the mechanical and drying properties of the film favorably. Silky on the skin, but neither shiny nor greasy [29].
Packaging material type	Well closing Appropriate for the dosage form	The system includes volatile components. The packaging material type is important to keep these volatile components in the formulation [15].
Mechanical properties of film for skin application	Flexible, highly adherent, resistant film	In order to ensure that the composition achieves the desired effect, the in situ formed film needs to have suitable mechanical properties, such as to stay on the skin constantly, to have flexible movement similar to that of the skin and to be easily removable in one piece [28].

**Table 3:** Critical quality attributes (CQAs) of the FFS.

CQA	Target	Justification
**Semisolid System Properties**		
Physical properties (color, odor, appearance)	Translucent or white appearance, homogeneous, clear and odorless smell	To increase patient compliance.
Viscosity	Optimal spreadability on the skin (range: 50,000–150,000 mPas)	Rheological attributes, e.g., viscosity, to influence the application of the formulation on the skin and the stability of the semisolid system [28].
Homogeneity	Homogenous distribution of the components in the formulation	Homogeneity ensures stability and aesthetic appearance. During application the uniformity of dosage units has to be maintained.
pH	Optimal pH of transdermal formulation (range: 4–8 pH)	For the safety and efficacy of the product.
Skin feeling	Not sticky, not greasy, silky touch on the skin	Formulations containing silicones influence skin feeling. They are slightly slippery and silky on the skin. Due to the semi-occlusive effect of silicones, the skin could be softer and well-hydrated [29].
Drying time	Optimal drying time for comfortable use (within 10 min)	The optimal drying time is a critical point of comfortable use. The formulation has to dry fast to avoid smearing [28].
Stability (physical, chemical)	No visible sign of instability at the time of preparation and after one month (at room temperature)	The physical and chemical stability of the semisolid system is essential to form a homogeneous, aesthetic appearance, and these properties ensure the mechanical attributes of the film for skin application.
Stability (microbiological)	Meets the requirements of the pharmacopoeia for dermally-applied systems	The safety of the FFS is a requirement for marketing authorization.
**Film properties**		
Film appearance	Translucent, homogeneous, compact film	To increase patient compliance (almost invisible, not shiny, easy to remove) [7].
Film burst strength	Compact film structure approaches the properties of the heat-separated human epidermis (range: under 5 N)	The film has to be strong enough to form a compact film on the skin and not to tear when the skin is moving.
Skin adhesion	Approach the adhesion of an adhesive plaster to the skin. (Mean peel range: 100–500 mN)	Good adhesion ensures the residence time on the skin for the appropriate exposure time.
Film flexibility	Approach the properties of the heat-separated human epidermis (range: above 25%)	To follow the skin moving, thereby avoiding the separation from the skin surface [28].
Film integrity	Compact film on the skin surface	To provide aesthetic appearance and easy removability. It can be pulled down completely [28].

**Table 4:** Summary of all the parameters that affect the FFS.

QTPPs	Impact	CQAs	CPPs and CMAs	Occurrence
Route of administration	High	Physical properties	Mixing rate	Medium
Dosage form	High	Viscosity	Mixing time	Low
Site of activity	Medium	Homogeneity	Type of mixer	Medium
Appearance of semisolid system	Medium	pH	Temperature of technology	High
Stability	High	Skin feeling	Type of technology	High
Silicone content	High	Drying time	Viscosity enhancing excipients	Medium
Type of packaging material	Medium	Stability (physical, chemical)	Preservatives	Low
Mechanical properties of film for skin application	High	Stability (microbiol.)	Drying excipients	High
		Film appearance	Film-forming excipients	High
		Film burst strength	Type of silicones	High
		Skin adhesion		
		Film flexibility		
		Film integrity		

**Table 5:** Risk estimation matrix of QTPP and CQA parameters (LeanQbD™ Software) Low = low risk, Medium = medium risk, High = high risk parameters during the research work.

	QTPP	Route of Administration (H)	Dosage Form (H)	Site of Activity (M)	Appearance of Semisolid System (M)	Stability (H)	Silicone Content (H)	Type of Packaging Material (M)	Mechanical Properties of Film for Skin Application (H)
CQAs									
Physical properties	5%	Low	Low	Low	High	High	Medium	Medium	Low
Viscosity	6%	Medium	Medium	Low	Medium	Medium	Medium	Medium	High
Homogeneity	6%	Low	High	Low	High	High	Medium	Low	Low
pH	5%	High	Medium	Medium	Low	Medium	Low	Low	Low
Skin feeling	5%	Medium	Medium	Low	Low	Low	High	Low	Medium
Drying time	9%	Medium	High	High	Low	Medium	High	Medium	High
Stability (physical, chemical)	7%	Low	High	Low	Medium	High	Medium	Medium	Medium
Stability (microbiol.)	5%	Low	High	Low	Medium	High	Low	Low	Low
Film appearance	10%	Medium	High	Low	Medium	High	High	Low	High
Film burst strength	11%	High	High	Low	Low	High	High	Low	High
Skin adhesion	11%	High	High	Medium	Low	High	High	Low	High
Film flexibility	11%	High	High	Medium	Low	High	High	Low	High
Film integrity	9%	Low	High	Medium	Low	High	High	Low	High

**Table 6:** Risk estimation matrix of CPPs/CMAs and CQA parameters (LeanQbD™ Software) Low = low risk, Medium = medium risk, High = high risk parameters during the research work.

	CMAs, CPPs	Mixing Rate (M) CPP	Mixing Time (L) CPP	Type of Mixer (M) CPP	Temperature of Technology (H) CPP	Type of Technology (H) CPP	Viscosity Enhancing Excipients (M) CMA	Preserv-Atives (L) CMA	Drying Excipients (H) CMA	Film-Forming Excipients (H) CMA	Type of Silicones (H) CMA
CQAs											
Physical properties	5%	High	Medium	High	High	High	High	Low	Medium	High	Medium
Viscosity	6%	High	Medium	Medium	High	High	High	Medium	Medium	Medium	Medium
Homogeneity	6%	High	High	High	High	High	High	Low	Medium	High	High
pH	5%	Low	Low	Low	Low	Low	High	Low	Low	Low	Low
Skin feeling	5%	Low	Low	Low	Low	Low	Medium	Low	High	High	High
Drying time	9%	Low	Low	Low	Low	Low	Medium	Low	High	Medium	High
Stability (physical, chemical)	7%	High	High	High	High	High	High	Low	Medium	High	High
Stability (microbiol.)	5%	Low	Low	Low	Low	Low	Low	High	Low	Low	Low
Film appearance	10%	Low	Low	Low	Low	Medium	Medium	Low	High	High	High
Film burst strength	11%	Low	Low	Low	Low	Low	Medium	Low	High	High	High
Skin adhesion	11%	Low	Low	Low	Low	Low	Medium	Low	High	High	High
Film flexibility	11%	Low	Low	Low	Low	Low	Medium	Low	High	High	High
Film integrity	9%	Low	Low	Low	Low	Low	Medium	Low	High	High	High

**Table 7:** DSC data of different FFSs.

FFS	Onset (°C)	Peak (°C)	Endset (°C)
F1	93.71	107.01	140.21
F2	59.16	71.70	80.02
	91.57	107.62	113.15
F3	90.67	91.54	93.77
	109.34	112.62	120.91

**Table 8:** Summary of the investigation of the film formed by FFSs; ✔✔: exceptionally good result, ✔: The result meets the requirement of Table 3, ✖: The result does not meet the requirement of Table 3.

	Formulation	F1	F2	F3
Investigation				
Skin adhesion		✔	✔	✖
Film flexibility		✔	✔	✖
Film burst strength		✔	✔	✔
Film appearance		✔	✔	✔
Film integrity		✔	✔	✔
Drying time		✖	✔	✔✔
